# The Conservative Management of the Carpometacarpal Joint Fracture-Dislocation: A Case of the Second to the Fifth Ray

**DOI:** 10.7759/cureus.75354

**Published:** 2024-12-09

**Authors:** Dimitrios Kalatzis, Konstantinos Zygogiannis, Konstantinos Kaoullas, Georgios Zoumpoulis, Georgios C Thivaios

**Affiliations:** 1 Orthopedics and Traumatology Department, Laiko General Hospital of Athens, Athens, GRC; 2 Scoliosis and Spine Department, KAT General Hospital of Athens, Athens, GRC; 3 Orthopedic Department, Laiko General Hospital of Athens, Athens, GRC

**Keywords:** carpometacarpal joint dislocation, complications, conservative and surgical treatment, diagnostic and therapeutic techniques, upper extremity fracture

## Abstract

Carpometacarpal (CMC) joint fractures-dislocations are rare due to the complex structure of the carpal bones and strong ligamentous support; while the clinical image is usually "noisy," they present significant management challenges due to the unstable nature of the injury. These injuries are typically caused by high-energy trauma and frequently result in dorsal dislocations. Treatment requires a careful balance between the immobilization and surgical restoration of the anatomical alignment to prevent complications. This report highlights a case of four CMC joint dislocations managed conservatively, yielding positive outcomes despite the usual indications for surgical intervention, avoiding any possible anesthesiological or surgical complications.

## Introduction

Carpometacarpal (CMC) joint fractures-dislocations are rare compared to other hand injuries given the intricate form of the carpal bones and the robust ligamentous structures that typically prevent dislocation [1]. The CMC joints are inherently stable due to their articulating surfaces and the supporting system of dorsal, volar, and intra-articular ligaments. By contrast, the trapeziometacarpal joint allows polyaxial movement in several planes because of its biconcavo-convex structure, yet this movement is made possible by the capsule and ligamentous structures that assist in stability. Thus, high-energy trauma, such as motor vehicle accidents or falls from significant heights, is usually required to produce enough force to dislocate these joints [2]. Among these injuries, dorsal dislocations are the most common presentation [3]. However, diagnosing CMC dislocations can be challenging for physicians, particularly in emergency settings, due to their subtle presentation, leading to frequent misdiagnosis [4]. Handling these injuries requires a careful balancing act. While immobilization is essential for the proper healing of the soft tissues for both surgical and conservative management, prolonged immobilization for more than 5-6 weeks can lead to complications such as joint stiffness, muscle weakness, and tendon adhesions [3]. In this regard, we report a case of four CMC joint dislocations treated conservatively despite the need for surgical stabilization, with a successful outcome.

## Case presentation

**Figure 1:**
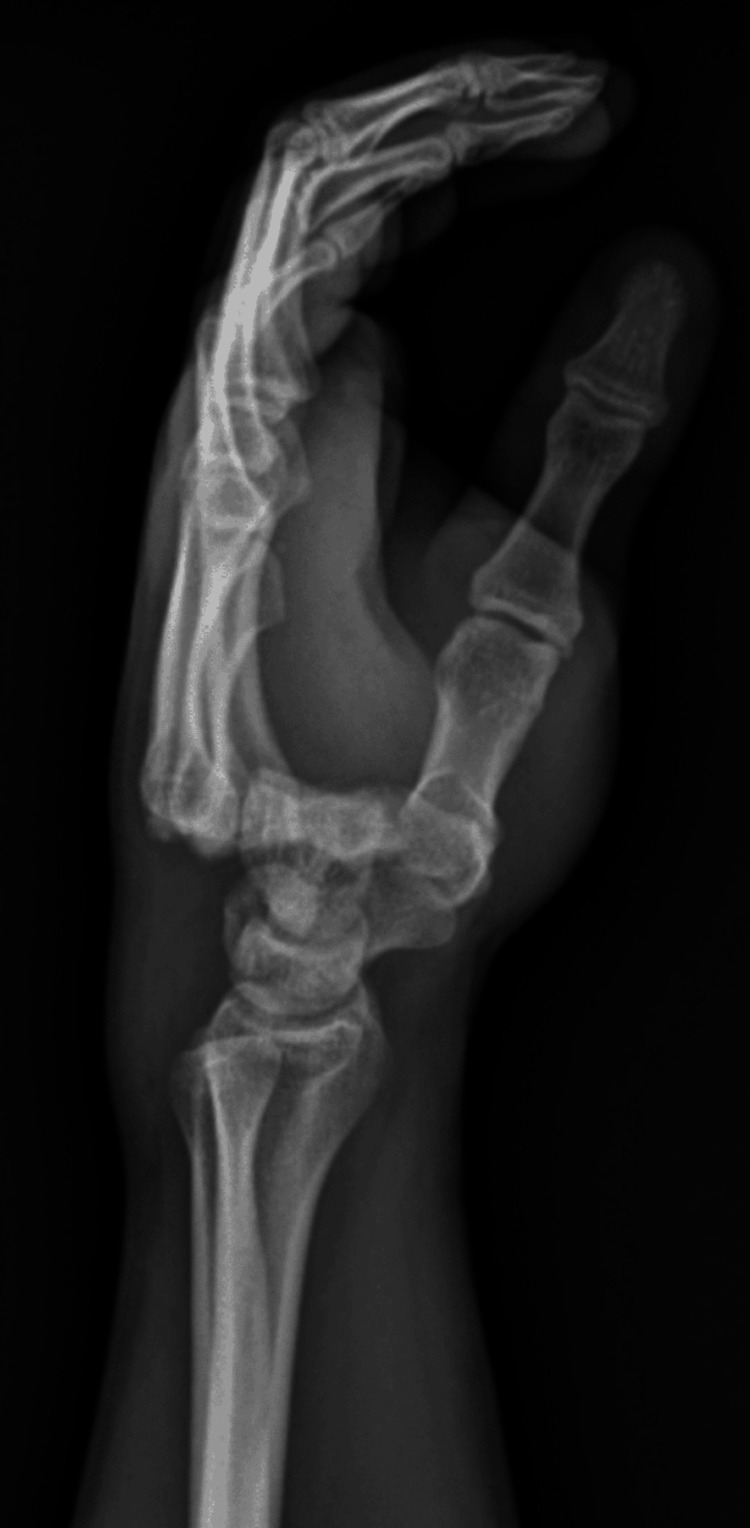
Lateral X-ray view of the carpometacarpal joint demonstrating dorsal dislocation

**Figure 2:**
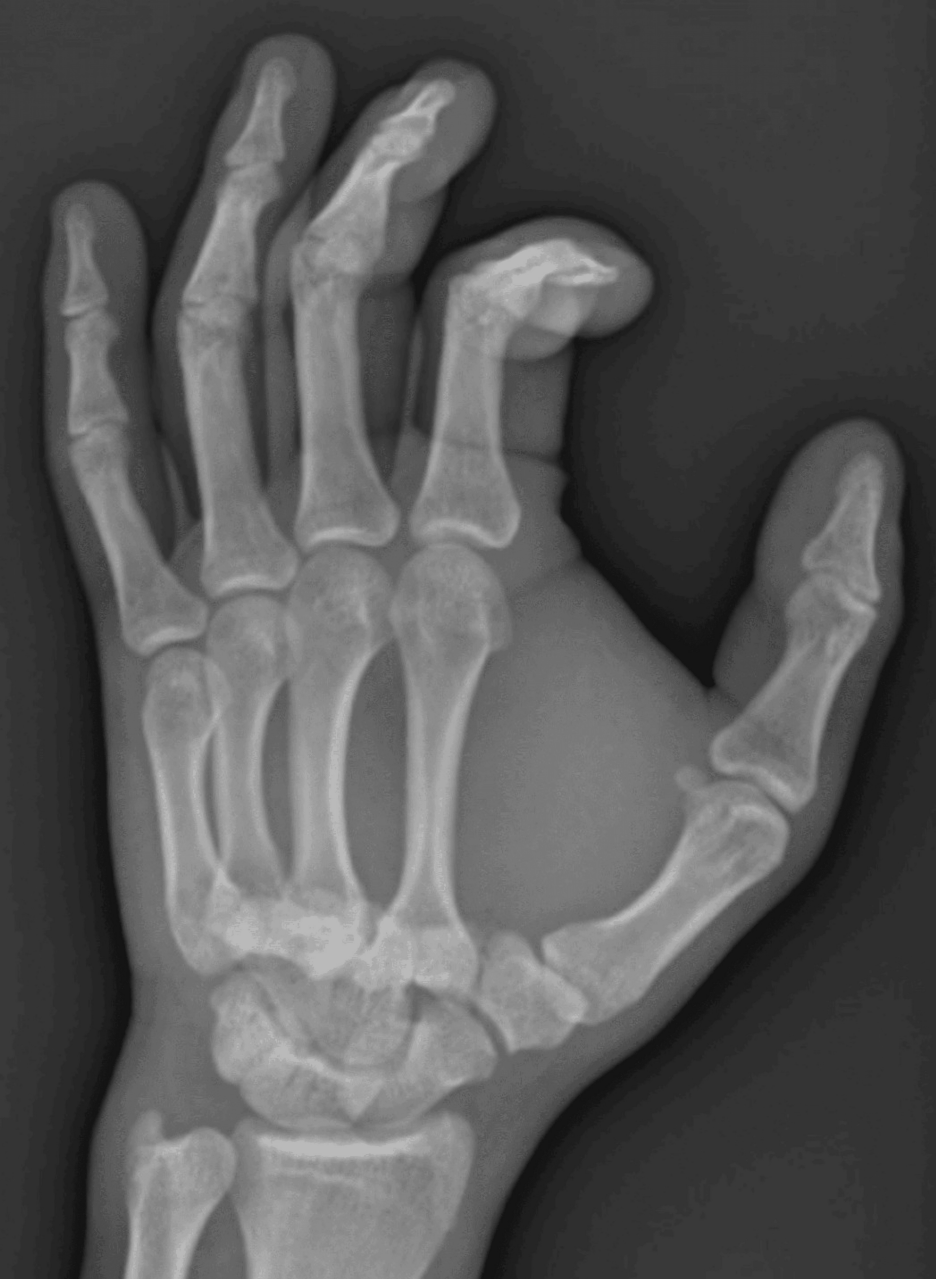
Anteroposterior view of the carpometacarpal joint with a loss of normal anatomy as the base of the metacarpal bones is not clearly seen

**Figure 3:**
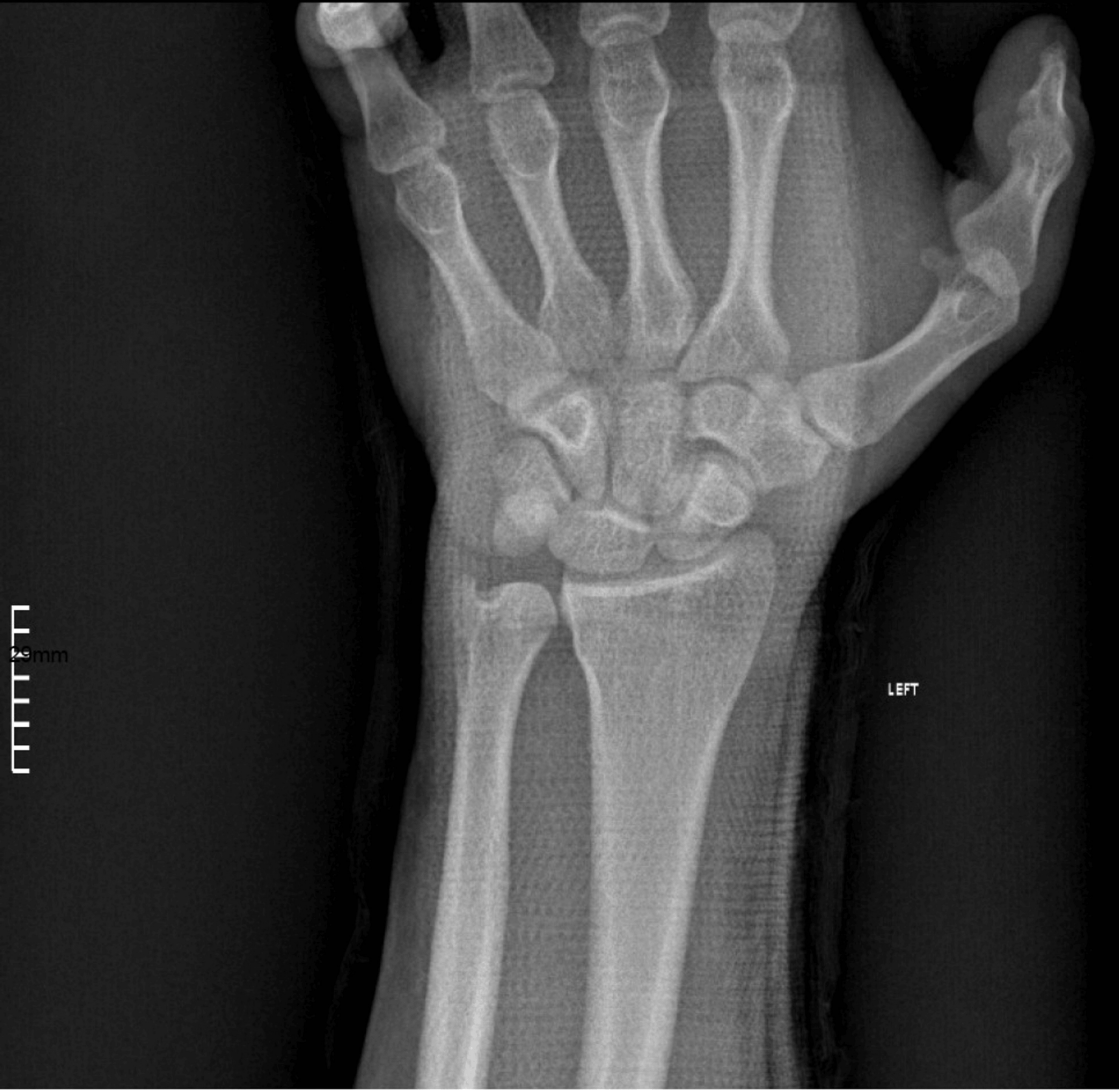
Post-reduction anteroposterior X-ray view of the carpometacarpal joints where the basis is clearly seen

**Figure 4:**
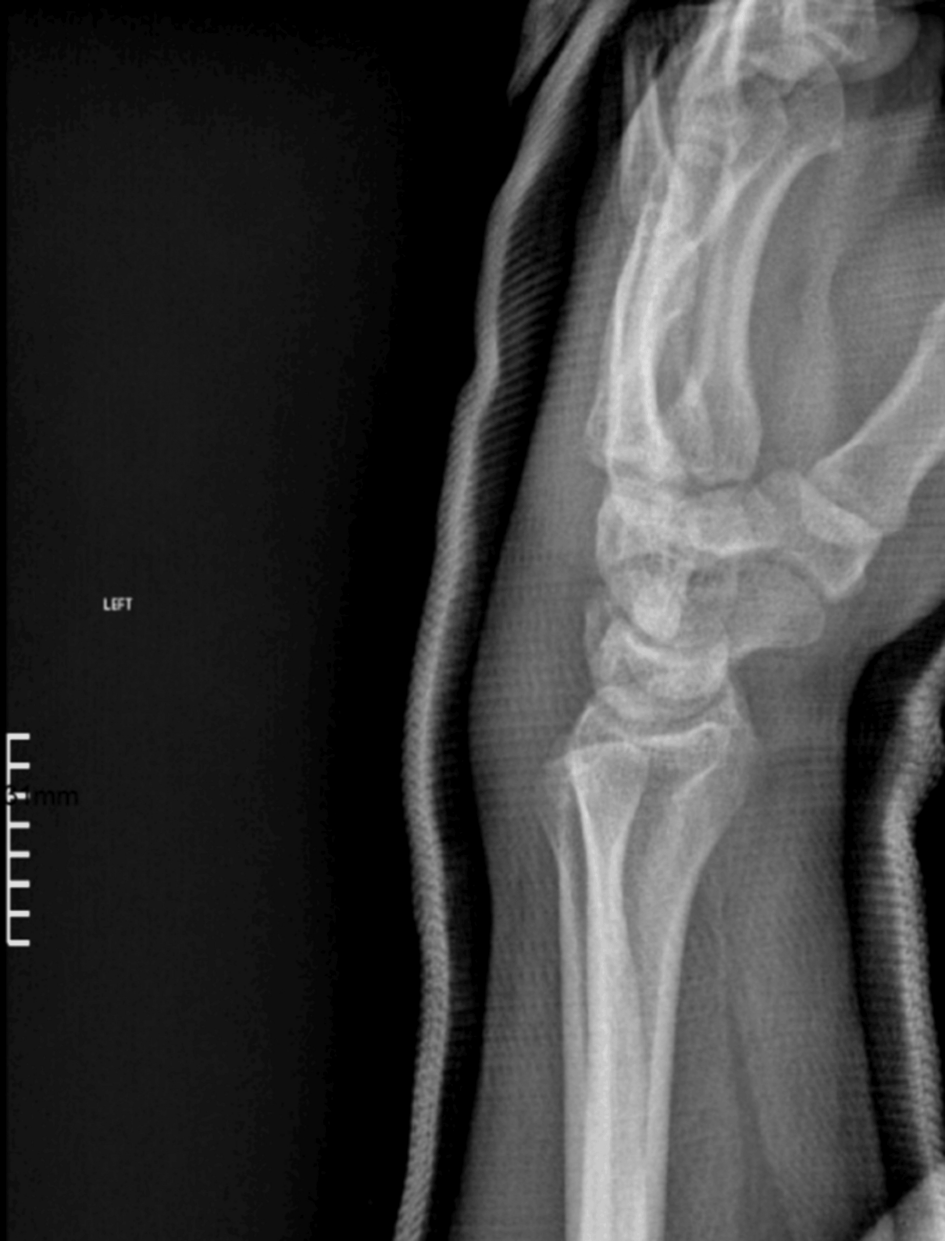
Post-reduction lateral X-ray view of the carpometacarpal joints

**Figure 5:**
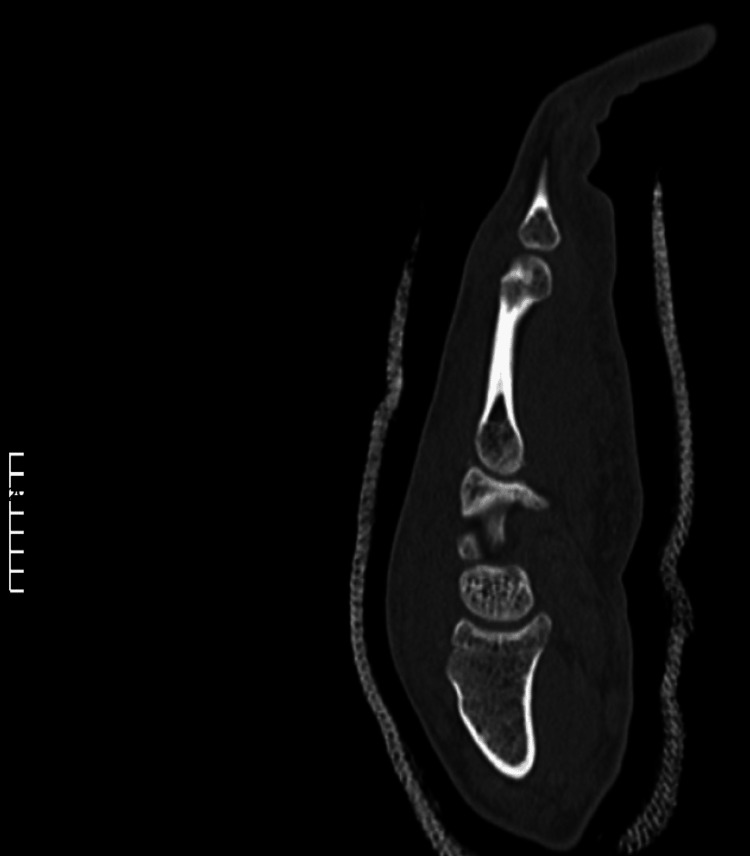
Post-reduction lateral CT view of the carpometacarpal joint

**Figure 6:**
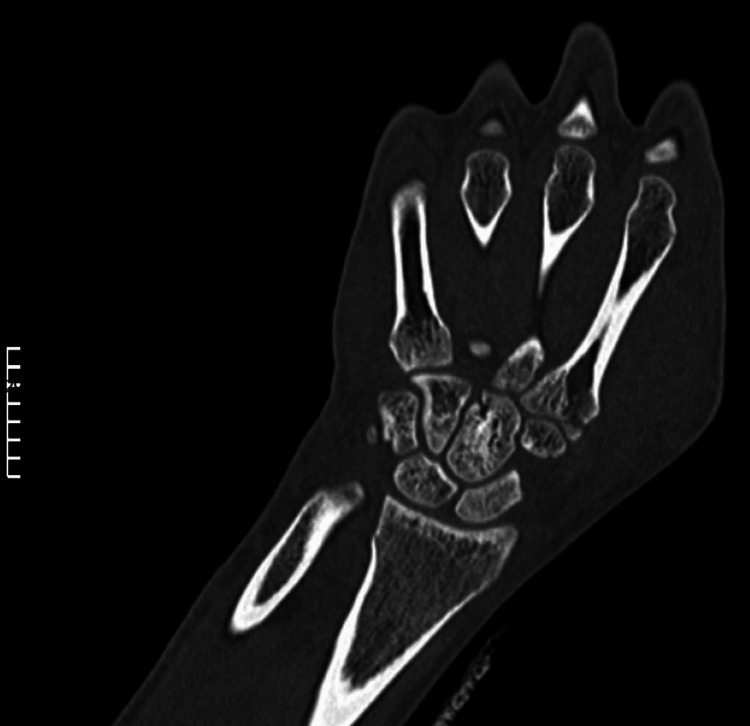
Anteroposterior view of the carpometacarpal joints demonstrating the reduction

A 22-year-old man presented at the emergency department after sustaining injuries from a motor vehicle accident. An obvious distortion of his nondominant hand, the left, was present. An examination revealed edema and step-off when palpating the hand distally to the wrist in the anatomical area of the CMC joints. No other injuries were recorded. The mechanism of injury, as the patient described, was a fall on an outstretched hand, with a violent force applied from the palmar to the dorsal at the metacarpal area. After radiological examination, the diagnosis of dorsal dislocation of the second, third, fourth, and fifth CMC joints was made (Figures 1, 2). Closed reduction by local anesthesia was achieved by applying a traction force to the fingers combined with a palmarly directed force at the base of the metacarpals. The loss of the palpable step-off confirmed the reduction, and the passive flexion-extension of the fingers revealed that the CMC joints were stable. Afterward, the wrist and hand were immobilized in a neutral position using both palmar and dorsal slabs, and X-rays and CT were made to confirm the reduction, obtain a more detailed imagistic assessment, and reveal any concomitant fractures (Figures 3, 4, 5, 6). A few days later, an MRI scan was ordered to establish the soft tissue injury and a baseline for the bone marrow edema.

A small fracture fragment of both the second metatarsal base and the capitate was identified. The patient was admitted to the orthopedic clinic for 24 hours, and close observation for compartment syndrome was made. Since the patient did not consent to surgical management and the dislocation of the CMC joints was found to be stable, we managed the patient conservatively. Regular follow-up with X-rays at weeks 1, 2, 3, and 6 was made to ensure no re-dislocation. The below-elbow splints were maintained for a total of six weeks. Afterward, hand therapy focusing on regaining the range of motion (ROM) was initiated using a circumferential carpal stabilization brace. The brace was utilized during hand therapy for another four weeks. Strengthening exercises started at 12 weeks. A follow-up of the patient was made at three, six, nine, and 12 months. He had regained full ROM and presented grip strength of 75% versus the contralateral side, using measurements from a hand dynamometer. The patient's occupation was an electrician's assistant, and he reported no pain during everyday activities and hand functions related to work at the final evaluation.

## Discussion

The carpometacarpal joints are incongruous and have only one degree of free motion. Specifically, the range of motion of the index, middle, ring, and little finger consists of five, 10, and 15 degrees of flexion, respectively [1]. However, the fifth CMC joint can be classified as a semi-saddle joint, allowing limited rotational movement. Thus, the fourth and fifth rays are more mobile, making cupping of the hand possible. Strong ligamentous attachments and the anatomy of the carpal bones render the dislocation of the CMC joints a rare injury [3]. Interesting is the fact that when attempting closed reduction, this was not made possible when starting from the second CMC joint. Only when trying to reduce firstly the fifth CMC joint, a reduction of all the CMC joints was then achieved. This fact might correlate with the anatomy of the fifth CMC joint as noted before [1]. After the reduction of the fifth ray, possibly, a domino effect was initiated due to the ligamentous attachments between the metacarpals, reducing the CMC dislocations easier. Numerous case reports and small retrospective studies assess mainly surgical management and postoperative outcomes without any reference to cases of nonsurgical treatment.

Kalinterakis et al. reported a case of a patient with third to fifth CMC dislocation associated with hamate fracture caused by an unusual mechanism of injury, which was treated successfully with open reduction and fixation with Kirschner wires. At six-month follow-up, the patient reportedly had excellent results [5]. Another case of a CMC joint fracture-dislocation was reported by Bell et al., caused by a vehicle collision. Initially, an unsuccessful closed reduction was attempted, followed also by an open reduction through a dorsal transverse incision and fixation with Kirschner wires [6]. Reportedly, the wires were removed after eight weeks of cast immobilization and passive hand motion, followed by intensive physiotherapy due to rigidity. A full range of motion was achieved 10 weeks after the cast removal. In a retrospective study of 15 patients, Gülabi et al. compared the surgical outcomes of close reduction and percutaneous pinning (CRPP) with open reduction and percutaneous pinning (ORPP). The mean visual analog scale (VAS) scores for the ORPP and CRPP groups were 2.33±0.50 and 1.67±0.52, respectively. The mean Quick Disabilities of the Arm, Shoulder, and Hand (Q-DASH) scores for the ORPP group were 13.63±3.21, while the CRPP group had an average of 9.05±2.36. Additionally, the mean grip strength values were 65.78±3.70 for the ORPP group and 75.17±6.11 for the CRPP group. Statistically, the ORPP group had significantly higher VAS and Q-DASH scores compared to the CRPP group, whereas the CRPP group demonstrated a significantly greater mean grip strength than the ORPP group [7]. In our case, due to the patient's refusal to proceed with surgical management, he was treated conservatively. He regained full ROM and satisfactory function of the hand nine weeks after the cast removal, enabling him to return to his former occupation and everyday activity.

## Conclusions

The article emphasizes that while the gold standard for a successful treatment for multiple CMC dislocations is surgical stabilization, further evidence is required to demonstrate that conservative treatment can remain a potential option in case the patient declines surgery. Conservative treatment requires prolonged immobilization, which can often cause stiffness, edema, and complex regional pain syndrome. Close follow-up is necessary to assess the progress and ascertain that we do not have a new dislocation due to the unstable nature of the injury. Immediate mobilization, once we consider the injury stable after the treatment, is necessary to promote functional recovery, especially in young patients. Rehabilitation typically involves progressive range-of-motion exercises, strengthening, and functional training once the splint or cast is removed. On the other hand, in cases where conservative treatment results in a new dislocation or reduced range of motion, surgical intervention may ultimately be a one-way road to a successful outcome. Therefore, a thorough discussion is necessary between the surgeon and the patient to carefully explain the risks of a conservative treatment.
